# T2-weighted cardiac magnetic resonance image and myocardial biomarker in hypertrophic cardiomyopathy

**DOI:** 10.1097/MD.0000000000020134

**Published:** 2020-06-05

**Authors:** Shi Chen, Liwei Huang, Qing Zhang, Jie Wang, Yucheng Chen

**Affiliations:** a Department of Cardiology, West China Hospital, Sichuan University; b Department of Cardiovascular Ultrasound and Noninvasive Cardiology, Sichuan Academy of Medical Sciences and Sichuan People's Hospital, Chengdu, Sichuan, China.

**Keywords:** cardiac magnetic resonance, high-sensitivity cardiac troponin T, hypertrophic cardiomyopathy, myocardial injury, T2-weighted imaging

## Abstract

The phenomenon of high signal intensity on T2-weighted imaging of cardiac magnetic resonance in hypertrophic cardiomyopathy (HCM) has been previously studied. However, the underlying histopathologic mechanism remains unclear. Elevated cardiac troponin can be detected in some HCM patients. A reasonable hypothesis is that high myocardial T2 signal is a potential marker of myocardial injury in HCM. We sought to investigate the association between cardiac troponin and the extent of high T2 signals in HCM patients.

Forty-four HCM patients underwent 3.0T cardiac magnetic resonance scanning. On T2-weighted images, the number of segments with high-signal intensity (myocardium-to-skeletal muscle signal intensity ratio >2) and the percentage of high-signal area (>2 standard deviation above the remote tissue) were measured in 16 myocardial segments along the LV mid-myocardial circumference on 3 short-axis images. The level of high-sensitivity cardiac troponin T (hs-cTnT) was also assessed.

Myocardial high T2 signals were identified in 33 (75%) patients and 144 (20.5%) segments. Elevated hs-cTnT was observed in 28 (63.6%) patients. The Cochran–Armitage test showed a statistically significant trend of increasing levels of hs-cTnT with elevated number of segments with myocardial high T2 signal (*P* = .002). Further, the percentage of myocardium with high T2 signal was significantly associated with the hs-cTnT level (Pearson correlation: *r* = 0.388, *P* = .009).

Myocardium with high T2 signals was very common in patients with HCM.Its extent is related with the level of plasma hs-cTnT.

## 1 Introduction

Hypertrophic cardiomyopathy (HCM) is characterized by varying degrees of myocardial hypertrophy and is caused by mutations in genes encoding sarcomeric proteins.^[1,2]^ It is a complex cardiovascular disorder, because the pathways from mutation to clinical phenotype are still unclear. Moreover, the clinical course of the disease has a large inter- and intrafamilial heterogeneity,^[3,4]^ in that patients with the same mutation can range from showing mild symptoms of heart failure late in life to the onset of sudden cardiac death at a young age. Cardiac magnetic resonance (CMR) is unique in its high spatial and temporal resolution with excellent contrast between blood pool and the myocardium, without the limitation of either imaging window or imaging plane.^[5–7]^ Therefore, there has been a recent increase in the use of CMR in HCM assessment.^[8–13]^ The increasing incorporation of CMR in clinical practice continues to improve our understanding of the subtle morphologic differences and their prognostic implications in HCM.^[8–10]^ Recently, a few researchers have reported the presence of myocardial high T2 signal in some HCM patients.^[11–13]^ However, its pathologic basis and clinical significance remain unclear. It has been proved that T2-weighted hyperintense regions delineate the ischemic myocardium after acute myocardial infarction.^[14]^ It is thus reasonable to hypothesize that high T2 signal is a marker of myocardial injury in HCM. Elevated cardiac troponin can be seen in patients with HCM,^[15,16]^ which suggests a possible underlying injury in HCM. Therefore, the aim of the present study is to investigate the relationship between myocardial high T2 signal and cardiac troponin T levels in HCM patients, to clarify whether T2-weighted imaging can reflect myocardial injury in HCM.

## 2 Methods

### 2.1 Study population

The patient cohort consisted of 44 consecutive patients referred to our hospital between January 2012 and December 2013. The diagnosis of HCM was based on a 2-dimensional echocardiogram identifying left ventricular hypertrophy (wall thickness of at least 15 mm) not associated with another cardiac or systemic disease capable of producing hypertrophy of an appropriate magnitude. We excluded patients with systemic hypertension, coronary artery disease, primary valvular heart disease, electronic ventricular pacing, prior cardiac surgery, prior septal ethanol ablation, and renal failure; additionally, patients with claustrophobia were also excluded, as they would be unable to tolerate CMR scanning.

For each patient, the clinical history including age, sex, symptoms, familial history, and medications was collected. Echocardiography was performed to measure the left ventricular outflow tract (LVOT) gradient with continuous-wave Doppler in the apical 5-chamber view. An LVOT obstruction was defined as a gradient measuring ≥30 mm Hg.

Blood samples were drawn early morning in the same day that the CMR scan were planned to perform from the antecubital vein of each subject after overnight fasting. Once drawn, the serum samples were centrifuged and immediately stored at -20°C until further analysis. An electrochemical luminescence instrument (e411; Roche, Basel, Switzerland) was used to test the specimens. The high-sensitivity cardiac troponin T (hs-cTnT) levels were measured using a commercially available assay (Roche Diagnostics, Mannheim, Germany). The limit of detection (LoD) was 5 ng/L (pg/mL), and values under this limit were routinely classified as 4.99 ng/L. According to the manufacturer, the upper reference limit (URL; the 99th percentile in a normal reference population) was 14 ng/L.

All patients gave their informed consent to participate in the study. This project was approved by the institutional ethic committee at West China Hospital of Sichuan University.

### 2.2 Cardiovascular magnetic resonance imaging

#### 2.2.1 MR protocols

CMR studies were performed with a 3.0-T MR scanner (Magnetom Tim Trio; Siemens Medical Solutions, Erlangen, Germany) using an eight-channel phased-array body coil. All images were acquired by ECG-gated breath-hold technique. T2-weighted dark blood turbo spin echo with spectral attenuated inversion recovery was applied to evaluate myocardial edema at short axis view of the LV (4 slices including basal, mid, distal LV, and 4 chamber view). The scanning conditions were as follows: TR, 1400 ms; TE, 68 ms; turbo factor, 17: slice thickness, 10 mm; matrix, 256 × 144 mm; and FOV, 300–340 mm. Steady-state free-precession cine images were acquired in 3 long-axes (2-, 3-, and 4-chamber), and consecutive short-axis views covered the LV from base to apex (TR: 3.4 ms, TE: 1.3 ms, flip angle: 50, FOV: 340 mm, matrix size: 256 × 144, slice thickness: 8 mm). Then, late gadolinium enhancement (LGE) images were acquired 10 to 12 minutes after intravenous administration of 0.15 mmol/kg of gadopentetate dimeglumine (Bayer Schering Pharma, Berlin, Germany) by using the inversion recovery technique in identical views (TR: 700 ms, TE: 1.56 ms, flip angle: 20, matrix: 256 × 144, TI was individually optimized to null normal myocardial signal using a TI-scout sequence).

#### 2.2.2 Image analysis

Images were analyzed using a dedicated CMR post-procession software (Qmass 7.6, Medis, Leiden, The Netherlands) by 2 cardiologists and a radiologist with experience in CMR imaging, whose joint opinion was reached by consensus. The endocardial and epicardial contours were manually drawn in short-axis views as obtained by an SSFP sequence. The end-diastolic volume, end-systolic volume, and left ventricular mass were measured. Left ventricular mass were indexed to height. Maximal LV end diastolic wall thickness was measured as previously described.^[17]^

The spatial distribution of high T2 signal and LGE was assessed in reference to the 16-segments model. To assess whether the LV segments were involved by myocardial edema, 5 regions of interest (ROI) were drawn in each left ventricular segment, and 5 regions of interests were drawn in the skeletal muscles (erector spinae muscle or lattissimus dorsi) with homogenous signal on the T2W images. The average signal intensity of each LV segment and skeletal muscle were calculated. The relative myocardial signal intensity was calculated as the ratio of each LV segment signal intensity and the skeletal muscle signal intensity. Consistent with previous studies, the cut-off value of an elevated relative myocardial signal intensity was set as 2.^[18,19]^ Those LV segments with elevated relative myocardial signal were considered as involved by myocardial edema. Next, the percentage of myocardium with high T2-signal intensity was quantified by delineation of the myocardium with signal intensity 2 standard deviations (SD) above the mean signal obtained in the remote normal myocardium. In PSIR images, to quantify the myocardium with LGE, a semiautomated gray-scale threshold technique was performed using 2 SDs above the mean of image intensities in a remote normal myocardial region in the same image as generally recommended.^[20]^

### 2.3 Statistical analyses

The data are presented as the mean ± SD for continuous variables or as percentages for categorical variables. Clinical characteristics were compared using the *t*-test for continuous variables. The relation between the number of segments with high T2 signal and hs-cTnT levels was assessed using with Cochran–Armitage test. The correlations between the percentage of myocardium with high T2 signal and hs-cTnT levels and the extent of LGE were examined using the Pearson test. Binary logistic regression analysis was performed to assess independent predictors of elevated hs-cTnT level in HCM patients. The dependent variables were dichotomized as follows: patients with elevated hs-cTnT (≥14 ng/L) were assigned a value of 1, whereas patients without elevated hs-cTnT (<14 ng/L) were assigned 0. Independent variables included percentage of myocardium with high T2 signal and LGE, left ventricular mass index, maximal left ventricular thickness, and the New York Heart Association (NYHA) functional classification, all of which were continuous. All probability values were for 2-tailed tests. *P* < .05 was considered statistically significant. Data processing and statistical analyses were performed using SPSS 17.0 software (SPSS, Chicago, IL).

## 3 Results

The basic clinical and echocardiographic characteristics of the 44 HCM patients are summarized in Table 1. The mean age was 50.9 ± 16.0 years, and 25 patients (55.6%) were male. Of the 44 patients, 2 (4.5%) had a family history of HCM, and 1 (2.3%) had a relative who suffered from cardiac sudden death. AF was paroxysmal in 4 patients and chronic in 4. The mean NYHA class was 2.4 ± 0.9 despite optimal medical therapy that consisted of beta blockers in 36 (81.8%) patients and calcium-channel blockers in 6 (13.6%). Eighteen patients had significant LVOT obstruction and mean baseline resting LVOT gradient. Serum hs-cTnT ranged from 3 to 362.1 ng/L (33.46 ± 57 ng/L). The hs-cTnT was significantly elevated (≥14 ng/L) in 28 patients (63.6%).

**Table 1:**
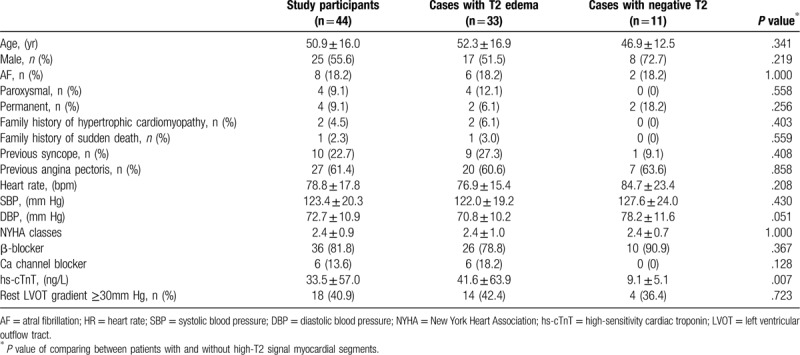
Clinical characteristics of study participants.

**Figure 1:**
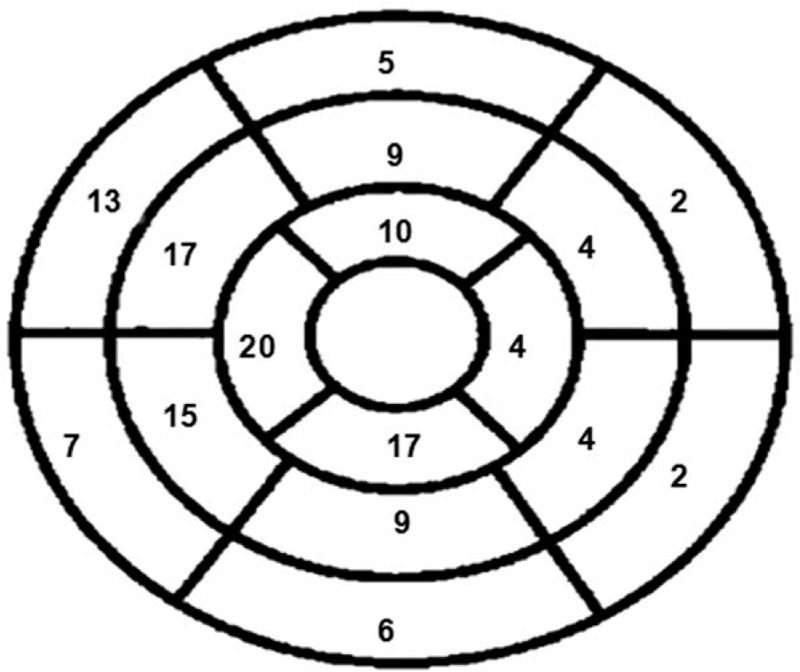
The distribution of a total of 144 segments of the high T2 signal is shown based on the 16-segment model with the total positive number in each segment.

**Figure 2:**
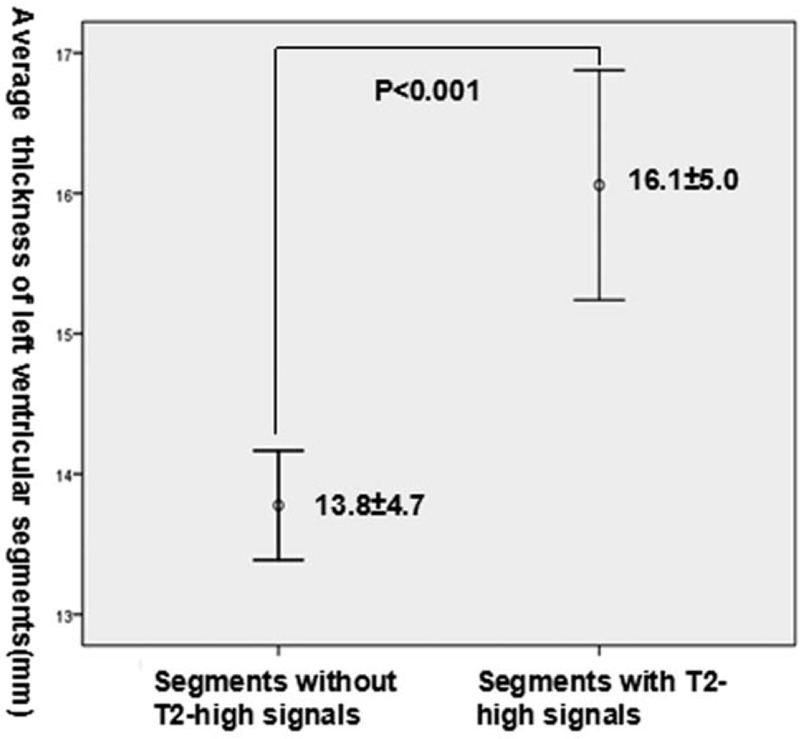
Comparison of the thickness of segments with and without high T2 signals in patients with hypertrophic cardiomyopathy.

Table 2 shows the CMR findings in our patients. Patients with HCM exhibited smaller LV end-systolic volume and LV end-diastolic volume . The LV wall was significantly thicker in HCM. The mean maximal LV wall thickness was 20.7 ± 5.7 mm. Elevated relative myocardial signal intensity (>2.1) was observed in 33 patients (75%) and 144 LV segments (20.5%). Figure 1 shows the distribution of the segments with elevated relative myocardial signal intensity for all our patients using the LV 16-segment model. Myocardial high T2 signal areas were most frequently identified in the ventricular septum at the basal to apical portion. The proportion of high T2 signal segments was lower in those segments adjacent to the septum and lowest in the remote segments farthest from the septum. Compared with segments with normal T2 signal intensity, Segments with high T2 signal intensity were significantly thicker than those with normal signal intensity (16.1 ± 5.0 vs 13.8 ± 4.7 mm, *P* < .001, Fig. 2).

**Table 2:**
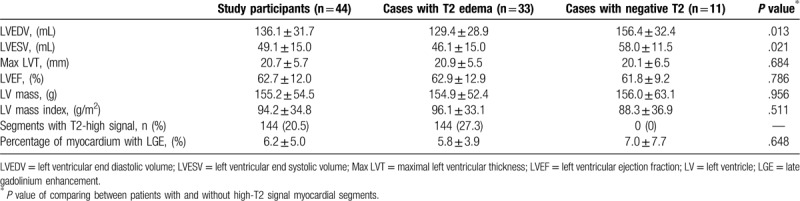
Cardiac magnetic resonance characteristics of patients with HCM.

**Figure 3:**
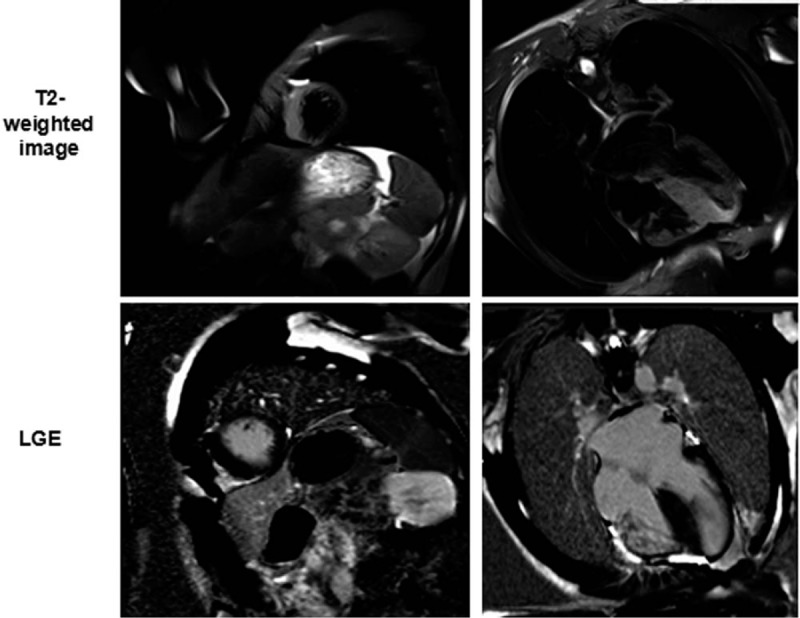
Cardiac magnetic resonance images of late gadolinium enhancement (LGE) and T2-weighted imaging in patients with hypertrophic cardiomyopathy. The LGE images (lower panel) and T2-weighted images (upper panel) are shown at the identical slice location in the same view. The areas of high T2 signal do not correspond well to those of LGE in patients with HCM.

From the subgroup analysis, we can see that the clinical and CMR characteristics were not different between patients with T2 edema and negative T2, except that patients with T2 smaller LV cavity and higher hs-cTnT levels (Table 1). To further explore the association between elevated T2 signal intensity, hs-cTnT levels and LGE, we performed a semi-quantitative analysis. We divided patients per tertile (number of segments with elevated T2 signal intensity; low: ≤1, mid: 2–4, high: ≥5) and examined the relation between the number of segments with high T2 signal, and hs-cTnT levels and the extent of LGE by using the Cochran–Armitage test. A statistically significant trend of increasing hs-cTnT levels with enlarging extent of high T2 signal was observed (*P* = .002). However, the relationship between the number of segments with high T2 signal and extent of LGE was not significant. This indicated that the areas of high T2 signal do not match well with those of LGE in HCM patients, as shown in Figure 3.

**Figure 4:**
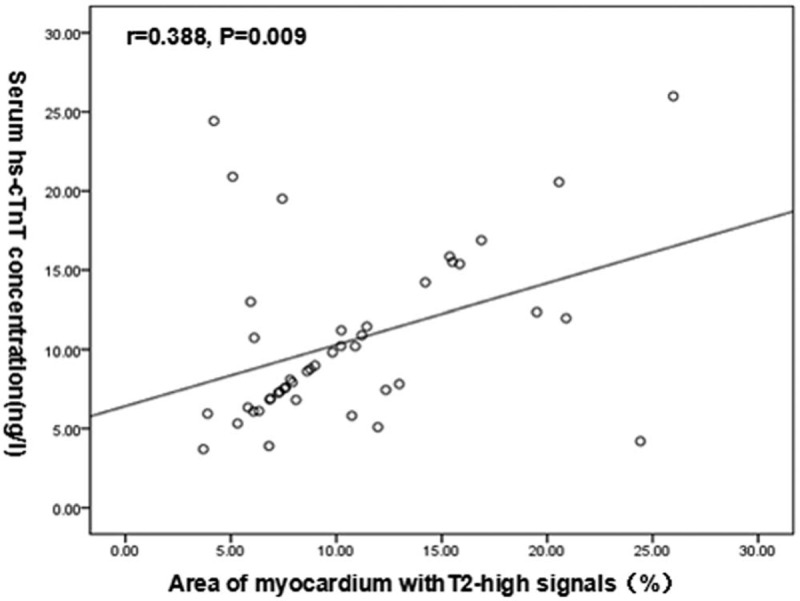
The relationship between the percentage of myocardium with high T2 signal and the concentration of serum high sensitivity cardiac troponin T (hs-cTnT) in patients with hypertrophic cardiomyopathy.

In the quantitative analysis, we calculated the percentage of myocardium with high T2 signal (2 SD above the signal intensity of remote normal myocardial tissue) in patients with T2 edema. Then, we used Pearson test to determine the relationship between percentage of myocardium with high T2 signal, hs-cTnT levels, and the extent of LGE to confirm the above results. Finally, we found that the percentage of myocardium with high T2 signal was significantly associated with the hs-cTnT level (*r* = 0.388, *P* = .009, Fig. 4); however, it was not correlated with the extent of LGE (r = 0.057, *P* = .418). Logistic regression analysis identified percentage of myocardium with high T2 signal as the only independent predictor of elevated hs-cTnT levels (OR: 0.704, 95%CI: 0.505–0.981, *P* = .038).

## 4 Discussion

To our best knowledge, this is the first report that provides data connecting the extent of myocardium with high T2 high signal and hs-cTnT level in HCM patients.

A large proportion of patients diagnosed with HCM have mutations in sarcomeric proteins. However, the mechanisms by which a mutation in the contractile proteins induces hypertrophy remain obscure. CMR has emerged as a particularly powerful imaging technique owing to its unique strengths of tomographic imaging and enhanced spatial resolution. Additionally, multiparametric MRI can provide detailed non-invasive tissue characterization of the myocardium. It may help us to clarify the course of HCM mutations developing in patients with left ventricular hypertrophy. Reports in recent years have described the presence of myocardium with hyperintense signals in T2-weighted CMR images in some patients with HCM.^[11–13]^ Consistent with the results of previous studies, in this group of 44 HCM patients with 704 left ventricular segments, myocardial high T2 signals were identified in 33 patients and 144 segments. We found that myocardial high T2 signal areas were more observed in the septum and adjacent segments that were mostly involved in HCM.^[21,22]^ Additionally, segments with high T2 signal were significantly thicker than those with normal T2 signal intensity.

The serum concentration of hs-cTnT is a novel and highly sensitive and specific indicator of subclinical or ongoing myocyte injury. Recent studies have reported that hs-cTnT was elevated in a significant number of HCM patients.^[15,16]^ Moreover, someone^[23]^ revealed that the hs-cTnT level was an independent predictor of cardiovascular events in patients with HCM. In this study of 44 HCM patients, 28 (64.6%) showed elevated hs-cTnT (>14 ng/L). Although the mechanisms of myocyte injury and release of hs-cTnT in HCM remain unresolved, we thought that they may be caused by relative myocardial ischemia or inflammation. Ischemia is a frequent find in HCM resulting from an imbalance between inappropriate hypertrophy of the myocardium and insufficient coronary arterial supply which appears to develop secondary to microvascular dysfunction, even in the absence of epicardial coronary stenosis.^[24]^ Frustaci et al^[25]^reported myocarditis, often viral, is not rare and represents a common cause of acute clinical deterioration in patients with HCM.

We found that the extent of myocardial high T2 signal was positively related with serum hs-cTnT levels. Our results indicated that high T2 signals are a marker of myocardial injury in patients with HCM. We speculate that high T2 signal may reflect a certain pathological alternation among involved myocardium in HCM. Signal intensity on T2-weighted MR images appears to be linearly related to myocardial water content. The common understanding is that myocardial high signal on T2-weighted imaging represents myocardial edema.^[26,27]^ However, a direct relation between the hydroxyproline-rich myocardial collagen and T2 relaxation time were also observed in the myocardium.^[28]^ Unlike in LGE imaging, wherein pathologic analysis shown by the precise shape and contour of the bright region exactly matches the area of myocardial scar,^[29]^ this level of validation does not exist for T2-weighted MR imaging in HCM. To determine the pathologic basis of T2-high signal, validation studies that provide a comparison between MR images and a pathology reference for the involved segments in HCM should be performed.

The prognostic impacts of myocardial high T2 signals in HCM patients is not clear. However, both Hen and Gommans found that the presence of myocardial high T2 signal is a significant independent predictor of life-threatening arrhythmia in HCM patients.^[11,12]^ It indicated that myocardial high T2 signals reflect disease progression and are associated with worse outcome in HCM patients.

LGE imaging offers the capability to directly visualize myocardial fibrosis in HCM. Hen^[11]^ observed that the high T2 signal areas were localized within the LGE areas in HCM patients. On the contrary, Amano^[13]^ found that high signal intensity of the myocardium on T2-weighted images often locates outside LGE. In our study, we found that the extent of LGE was not correlated with the extent of myocardium with high T2 signals. We speculate that in HCM, the involved myocardium undergoes temporal evolution, passing through an acute edematous stage with high T2 signals and ending with mature chronic fibrous tissue with no obvious T2 abnormalities. To confirm our hypothesis, a follow-up study should be performed.

This observational study found a significant association between elevated hs-cTnT and the range of myocardium with high T2-weighted signal intensity that provides supportive evidence for the hypothesis that high T2 is indicative of sustained cardiac injury and may cause acute clinical deterioration in HCM. So, HCM patients with high T2 should be given closer attention and clinic follow-up.For these patients some special treatment such as implantable cardioverter-defibrillators should be considered in earlier stage.

Our study has several limitations. The primary limitation is the cross-sectional study design. As such, we were unable to study the correlation between CMR characteristics and clinical outcomes. Second, T2-weighted imaging and semi-quantitative evaluation were used to analyze the extent of high T2 signals. Moreover, T2-weighted images are generally associated with problems including signal intensity variability caused by phased array coils, high signal from slow moving ventricular chamber blood that can mimic and mask elevated T2 signals in the sub-endocardial myocardium, and motion artifacts. To validate our results and better understand the prognostic impacts of our findings, we plan to perform a follow-up study with quantitative T2 mapping technique. Third, the sample size is relatively small given that HCM is a rare disease.Fourth, hs-cTnT is a biomarker which indirectly indicate myocardial injury. We did not investigate direct pathological evidence of myocardial high T2 signal.

## 5 Conclusions

Myocardium with high T2 signals was very common in patients with HCM. Although the underlying histopathological alternation is unclear, the extent of myocardium with high T2 is related with the level of plasma hs-cTnT that can be considered as a marker of myocardial injury.

## Author contributions

**Conceptualization:** Yucheng Chen, Shi Chen.

**Data curation:** Shi Chen.

**Formal analysis:** Shi Chen.

**Investigation:** Shi Chen.

**Methodology:** Shi Chen.

**Project administration:** Shi Chen.

**Software:** Liwei Huang.

**Supervision:** Yucheng Chen.

**Validation:** Yucheng Chen.

**Visualization:** Liwei Huang, Yucheng Chen.

**Writing – original draft:** Shi Chen, Liwei Huang.

**Writing – review & editing:** Yucheng Chen.
